# Cross-Corpus Speech Emotion Recognition Based on Transfer Learning and Multi-Loss Dynamic Adjustment

**DOI:** 10.1155/2022/5019384

**Published:** 2022-09-20

**Authors:** Huawei Tao, Yang Wang, Zhihao Zhuang, Hongliang Fu, Xinying Guo, Shuguang Zou

**Affiliations:** College of Information Science and Engineering, Henan University of Technology, Zhengzhou 450001, China

## Abstract

In this paper, we do research on cross-corpus speech emotion recognition (SER), in which the training and testing speech signals come from different speech corpus. The mismatched feature distribution between the training and testing sets makes many classical algorithms unable to achieve better results. To deal with this issue, a transfer learning and multi-loss dynamic adjustment (TLMLDA) algorithm is initiatively proposed in this paper. The proposed algorithm first builds a novel deep network model based on a deep auto-encoder and fully connected layers to improve the representation ability of features. Subsequently, global domain and subdomain adaptive algorithms are jointly adopted to implement features transfer. Finally, dynamic weighting factors are constructed to adjust the contribution of different loss functions to prevent optimization offset of model training, which effectively improve the generalization ability of the whole system. The results of simulation experiments on Berlin, eNTERFACE, and CASIA speech corpora show that the proposed algorithm can achieve excellent recognition results, and it is competitive with most of the state-of-the-art algorithms.

## 1. Introduction

Speech emotion recognition (SER) is an essential technical foundation for human-computer interaction. Traditional research on SER is often based on the same corpus for training and testing and has achieved excellent recognition performance. However, the feature distributions of the training and testing data vary greatly in practical application scenarios. Due to the different recording environments, personnel, gender, age, and languages of different corpus, the distribution of speech features from different corpus can be highly diverse, which is a typical cross-corpus SER problem [1]. Therefore, it is a very important research to deal with the differences in feature distributions for cross-corpus SER.

In the past decades, most of the speech emotion recognition algorithms were implemented under the setting that the training and testing sets belong to the same corpus, and researchers have achieved great success in this restricted experimental setup. Early research was carried out based on traditional machine learning methods, such as support vector machines (SVM) [2], Gaussian mixture models (GMM) [3], hidden Markov models (HMM) [4], K-Nearest Neighbor (KNN) [5], and other methods for processing speech features, and in recent years, with the development of deep learning, convolutional neural networks (CNNs) [6], recurrent neural networks (RNNs) [7], long short-term memory (LSTM) [8], deep belief networks (DBNs) [9], auto-encoders (AEs) [10], and other methods are applied to speech feature extraction, and these data-driven deep learning methods have obtained excellent performance improvement in SER tasks. Notably, these methods are also widely used in semantic sentiment analysis, with some studies [11, 12] using CNNs and AE to learn text feature representations, and these techniques, together with speech sentiment recognition, have driven the development of affective computing research.

As mentioned above, speech emotion recognition methods have gained unprecedented progress under restricted experimental conditions, based on which, the generalization enhancement of speech emotion recognition systems has gained more attention, which is the key to help the promotion of speech emotion recognition systems to real-world applications. Cross-corpus speech emotion recognition research has been conducted by setting the training and testing sets as different corpora to simulate the test data and training data in practical applications. Early cross-corpus SER research alleviates the problem of differences between corpora by manufacturing different acoustic feature sets and normalizing them for generalization. Existing research methods mostly use deep learning methods to extract domain invariant representations and reduce the differences in feature distributions between training and test corpora through metric learning methods to improve the model. To further enhance the generalization of the speech emotion recognition model, we first add noise to the original acoustic features, which can help the network to extract robust emotion features, and secondly, a fine-grained metric learning method is used to alleviate the negative migration in the global domain adaptation process, and the main contributions of this work can be summarized as follows.

- An auto-encoder is designed to perform both emotion classification and features reconstruction tasks, extracting sentiment information from features while preserving the discriminative properties of the original features.
- The combination of global and subdomain adaptive algorithms alleviates the negative transfer problem caused by mismatching between different subclasses.
- Loss weights are optimized using a dynamic weight adjustment algorithm, and additional hyperparameters are used to assign different initial weights to multiple losses to better suit emotion recognition tasks.
- We conducted six sets of cross-corpus speech emotion recognition experiments on three different speech corpora with multilingual and different cultural backgrounds, and the overall results were better than the state-of-the-art algorithms.

The rest of the paper is organized as follows: Section 2 provides an overview of related work on cross-corpus speech emotion recognition. In Section 3, the implementation of the proposed method is discussed in detail.  Section 4 describes the datasets and presents the details of our experimental settings. The experimental results and comparison of the proposed method with different methods are also presented in Section 4. Finally, in Section 5, we present the conclusions of our work.

### 1.1. Related Work

Over the past few years, many machine learning and deep learning techniques have been successfully applied to cross-corpus SER. Researchers who have carried out research based on machine learning methods have first searched for discriminative domain invariant representations through feature reduction or regression analysis and mitigated interdomain discrepancies using domain adaptive techniques to achieve cross-corpus speech emotion recognition. Zong et al. [13] proposed a Domain-adaptive Least Squares Regression (DaLSR) model which trains a least squares regression model by adding regularization constraints to the objective function, to mitigate differences between source and target domains. Liu et al. [14] built a projection matrix to map the source and target speech signals to a common subspace, so as to obtain similar feature distributions and improve the generalization of the classifier. Luo et al. [15] searched for a latent low-rank feature space by incorporating the label information of the training speech corpus into the nonnegative matrix factorization, to minimize the marginal distribution and conditional distribution differences between the two corpora simultaneously. Song [16] used the nearest neighbor graph algorithm to measure the similarity between different corpora in the common subspace; then, the feature grouping strategy is used to search the high transferable part of emotional features based on [16]. Zhang and Song [17] introduced a *ℓ*_1,2_ -norm penalty in constructing a common subspace to learn the common features of different corpora; in addition, a new nearest neighbor graph algorithm was used to measure the interdomain similarity. In [18], a linear regression model was established to learn the domain invariant regression matrix, by modifying the maximum mean discrepancy (MMD), and both the marginal and conditional probability distribution between domains were considered. Recent research [19] has also used ensemble learning to perform cross-corpus SER, using a multiple classifier voting approach to classify emotions, achieving recognition results beyond traditional machine learning methods.

Since machine learning and deep learning techniques have shown strong feature learning capabilities in many fields, such as image recognition [20, 21], smart city development [22, 23], network security [24, 25], and recent epidemic prevention and control [26, 27], various advanced deep learning models have also been used in cross-corpus SER. In [28], deep belief networks were used to achieve more advanced performance than sparse auto-encoder and SVM on five corpora of three languages. Zhang et al. [29] used a convolution neural network to process speech features, completed source corpus emotion classification, and created f-similarity preservation loss to maintain label similarity between source domain and target domain, which enhanced the robustness of the system. An unsupervised domain adaptation approach was used by Ahn et al. [30] to develop a robust emotion recognition model that learns class similarity based on rare sample data in the source domain and adapts it to the target domain. Chang et al. [31] minimized discrepancy in maximally distorted samples by adjusting the acoustic feature encoder and adversarial training, thus enhancing the semantic consistency in the source and target domains. Das et al. [32] proposed a variational auto-encoder with KL annealing and a semisupervised auto-encoder, which achieved comparable classification accuracy as the denoising auto-encoder and a more consistent latent embedding distribution over datasets.

The above traditional machine learning methods or deep learning algorithms have achieved competitive performance in cross-corpus speech emotion recognition tasks, but there are still some limitations that need to be addressed. Most methods only align feature distributions globally or locally, and some ignore the need to adjust the weights of different losses during the training process although they take both into account, which would decrease the generalization ability of the features during transfer, and the proposed multi-loss dynamic adjustment methods proposed in this paper could be a good solution to these problems, and the techniques used by related work as well as the corpus are listed in Table 1.

## 2. Methods

**Figure 1:**
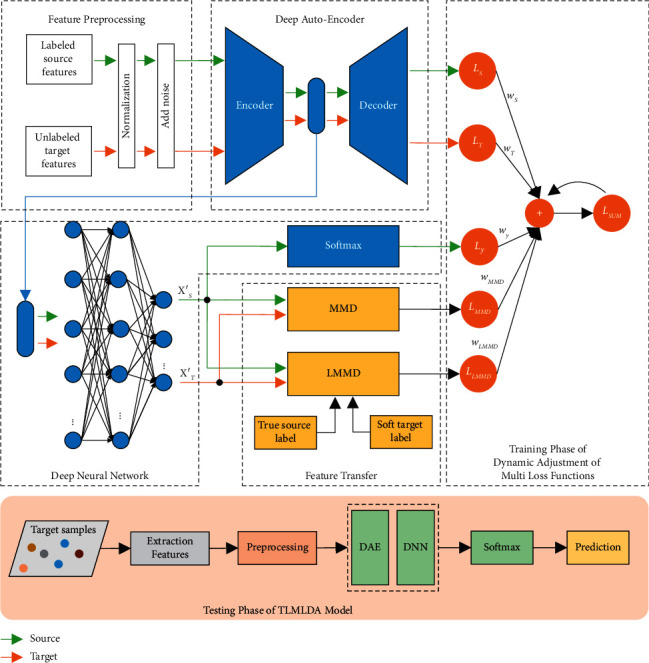
The TLMLDA model proposed in this paper. The flowchart above shows the training phase, and the flowchart below is the testing phase.

The general block diagram of the proposed model in this paper is shown in Figure 1. In the flowchart of training phase, the blue part represents the DAE and DNN structure. The yellow part indicates the joint MMD and local maximum mean discrepancy (LMMD) [33] for transfer learning. The orange part shows the multi-loss function dynamic adjustment, which uses dynamic weighting factors to adjust the importance of each loss function. In testing phase (below part of Figure 1), the target domain sample emotion features are processed by DAE and DNN, and subsequently, the SoftMax classifier is used for emotion classification.

### 2.1. Network Model

In the cross-corpus SER, the source domain samples features are **X**_*S*_=[**x**_1_^*S*^, ⋯, **x**_*n*_*s*__^*S*^] ∈ *ℝ*^*d*×*n*_*S*_^, the labels of the source domain samples are **Y**_*S*_=[**y**_1_, ⋯, **y**_*n*_S__] ∈ *ℝ*^*C*×*n*_*S*_^, and the target domain samples features are **X**_*T*_=[**x**_1_^*T*^, ⋯, **x**_*n*_*T*__^*T*^] ∈ *ℝ*^*d*×*n*_*T*_^, where *n*_*S*_ and *n*_*T*_ denote the number of samples in the source and target domains, respectively, *d* denotes the dimensionality of the emotion features of each speech sample, and *C* represents the number of emotion classes.

In order to obtain emotion features with strong representation, DAE is used to compress redundant information on features. Noise obeying normal distribution (with mean 0 and variance 1) is added to the source domain samples features and target domain samples. Then, noise features are input into the DAE. The features loss function of DAE consists of the reconstructed loss function *ℒ*_*S*_ of **X**_*S*_ and the reconstructed loss function *ℒ*_*T*_ of **X**_*T*_, which are denoted in the following equation:

(1) $$\begin{array}{c} L_{S}\left(X_{S},{\tilde{X}}_{S}\right)=\overset{n_{S}}{\underset{i=1}{\sum }}{\left\|x_{i}^{S}-{\tilde{x}}_{i}^{S}\right\|}^{2}\text{,}\\ L_{T}\left(X_{T},{\tilde{X}}_{T}\right)=\overset{n_{T}}{\underset{i=1}{\sum }}{\left\|x_{i}^{T}-{\tilde{x}}_{i}^{T}\right\|}^{2}\text{,} \end{array}$$

where ${\tilde{x}}_{i}^{S}$ and ${\tilde{x}}_{i}^{T}$ are the samples after DAE reconstruction.

As DNN is a nonlinear network structure, which can be approximated by complex functions and has a strong ability to learn the essential characteristics of data sets from a few sample sets, the encoded output of DAE is fed into the DNN network for processing to finally obtain the low-dimensional emotional features, which are **X**_*S*_′=[**x**′_1_^*S*^, ⋯, **x**′_*n*_*S*__^*S*^] ∈ *ℝ*^*d*′×*n*_*S*_^ and **X**_*T*_′=[**x**′_1_^*T*^, ⋯, **x**′_*n*_*T*__^*T*^] ∈ *ℝ*^*d*′×*n*_*T*_^. The source domain sample **X**_*S*_′ is predicted using the SoftMax classifier to obtain the probability value ${\bar{Y}}_{S}$, and then ${\bar{Y}}_{S}$ is cross-entropy calculated with the source domain true label **Y**_*S*_ to obtain the source domain classification loss *ℒ*_*y*_.

(2) $$\begin{array}{c} L_{y}\left(Y_{S},{\bar{Y}}_{S}\right)=\frac{1}{n_{s}}\overset{n_{s}}{\underset{i=1}{\sum }}y_{i}^{S}\;\log\;\left({\bar{y}}_{i}^{S}\right)\text{.} \end{array}$$

### 2.2. Feature Transfer

The MMD algorithm can be used to implement feature migration to reduce the difference between the source and target domains. In the low-dimensional emotional space of **X**_*S*_′ and **X**_*T*_′, the loss function of MMD is denoted in the following equation:

(3) $$\begin{array}{c} L_{MMD}\left(X_{S}^{\prime },X_{T}^{\prime }\right)={\left\|\frac{1}{n_{S}}\overset{n_{S}}{\underset{i=1}{\sum }}\delta \left({x^{\prime }}_{i}^{S}\right)-\frac{1}{n_{T}}\overset{n_{T}}{\underset{i=1}{\sum }}\delta \left({x^{\prime }}_{i}^{T}\right)\right\|}_{Η}\text{,} \end{array}$$

where Η is the reproducing kernel Hilbert space (RKHS) and *δ*(·) is the Gaussian kernel mapping function that maps the samples to the RKHS.

Considering the impact of subdomain mismatch, TLMLDA adopts LMMD to adjust the feature distribution of the emotion subdomain. LMMD divides the entire feature space into separate subdomain spaces according to emotion classes and adjusts the feature distribution between the source and target domains in the subdomain space. The loss function of LMMD is shown in the following equation:

(4) $$\begin{array}{c} L_{LMMD}\left(X_{S}^{\prime },X_{T}^{\prime }\right)=\frac{1}{C}{\overset{C}{\underset{c=1}{\sum }}\left\|\frac{1}{n_{S}}\overset{n_{S}}{\underset{i=1}{\sum }}\mu_{i,c}^{S}\delta \left({x^{\prime }}_{i}^{S}\right)-\frac{1}{n_{T}}\overset{n_{T}}{\underset{i=1}{\sum }}\mu_{i,c}^{T}\delta \left({x^{\prime }}_{i}^{T}\right)\right\|}_{Η}\text{,} \end{array}$$

where *μ*_*i*,*c*_^*S*^ and *μ*_*i*,*c*_^*T*^ are the weights of each sample belonging to one of the classes' emotion *C* in **X**_*S*_′ and **X**_*T*_′, respectively. The weights *μ*_*i*,*c*_ of the samples **X**′ are calculated as $\mu_{i,c}=/\underset{\left(\right)}{\sum }$. It is worth noting that the labels **Y**_*S*_^*C*^ of the source domain samples are known, while the target domain samples do not have label information and **Y**_*T*_^*C*^ cannot be calculated directly. Here, the labels **Y**_*T*_^*C*^ of the target domain samples **X**_*T*_′ are predicted by SoftMax.

### 2.3. Multi-Loss Optimization Training

TLMLDA transforms multiple loss functions into an overall loss function by weighted summation as

(5) $$\begin{array}{c} \min L_{sum}=\sum_{i}w_{i}L_{i}\text{,} \end{array}$$

where *i* ∈ {*S*, *T*, *y*, *MMD*, *LMMD*}, and *w*_*i*_ > 0 is the weighting factor of the loss function. There are great differences in the training speed of the five loss functions of TLMLDA, so the model has difficulty in obtaining the global optimal solution. To balance the optimization progress of each loss function, TLMLDA, motived by [34], constructs a dynamic weighting factor *w*_*i*_ to adjust the importance of the five loss functions. The dynamic weighting factor is

(6) $$\begin{array}{c} w_{i}=\frac{\alpha_{i}\times L_{i}}{L_{S}\left(X_{S},{\tilde{X}}_{S}\right)+L_{T}\left(X_{T},{\tilde{X}}_{T}\right)+L_{y}\left(Y_{S},{\bar{Y}}_{S}\right)+L_{MMD}\left(X_{S}^{\prime },X_{T}^{\prime }\right)+L_{LMMD}\left(X_{S}^{\prime },X_{T}^{\prime }\right)}\text{,} \end{array}$$

where *α*_*i*_ > 0 is a fixed hyperparameter, to strengthen the contribution of different losses in the overall loss according to experience. *ℒ*_*i*_ represents the value of loss functions *ℒ*_*S*_, *ℒ*_*T*_, *ℒ*_*y*_, *ℒ*_*MMD*_, and *ℒ*_*LMMD*_.

In the process of training, the TLMLDA model uses gradient descent algorithm, and a set of loss function values is generated at the end of each training. Then, the loss function values are used to update the *w*_*i*_ in Equation (5) to achieve dynamic adjustment of the loss weights.

## 3. Experiments Setup and Results Analysis

### 3.1. Data Preparation

We chose three public speech emotion corpus as cross-corpus SER corpora, which include Berlin [35], eNTERFACE [36], and CASIA [37]. Berlin is one of the widely used corpora in SER research, which contains anger, boredom, disgust, fear, happiness, sadness, and neutral emotions of 10 actors and a total of 535 speech samples. The eNTERFACE is a public audio-visual emotional corpus; it contains anger, disgust, fear, happiness, sadness, and surprise of 42 subjects from different nationalities and a total of 1287 speech samples. CASIA is a Chinese speech emotion corpus, which consists of 6 emotions (anger, fear, happiness, neutrality, sadness, and surprise) from 4 speakers with 1200 speech samples.

The experimental scheme is designed by selecting two speech samples with the same emotional label from the above three corpus, and one of the corpora is used as the source domain, and another corpus is used as the target domain. We designed six cross-corpus SER experimental schemes, E⟶B, B⟶E, E⟶C, C⟶E, B⟶C, and C⟶B, where B, E, and C are the abbreviations of Berlin, eNTERFACE, and CASIA, respectively. We summarize the speech sample labels and sizes used in these six cross-corpus SER schemes in Table 2.

### 3.2. Experimental Setup

We adopted the feature set of INTERSPEECH 2010 Paralinguistic Challenge [38], which contains 1582 dimensional features. Firstly, the feature set obtains 1428 dimensional features based on 34 low-level descriptors (LLDs) using 21 statistical functions. Secondly, based on the LLDs and Delta coefficients of the four pitch-based, 19 statistical functions are applied to obtain 152-dimensional features. In addition, the onset of pitch and durations of utterances are included into the feature set. Finally, the feature set obtained a total of 1582-dimensional features. Speech feature sets are extracted by the open-source openSMILE tool [39].

Under our experimental setup, speech features of source and target domain samples are normalized before input network training, where the range of each feature is scaled to the interval [0, 1] through Min-Max normalization. For DAE, the number of hidden layers is set to 6, and the sizes of the hidden layer neuron nodes are fixed to 1200, 900, 500, 900, 1200, and 1582, respectively. The activation function is set as ELU function in encoder phase and Sigmoid function in decoder phase. In addition, the batch normalization (BN) layer and dropout layer are also added to each layer structure of the DAE. For DNN, the number of hidden layers is set to 2, and the size of hidden layer neuron nodes of DNN is 600 and 256, respectively, and the activation function is set as Sigmoid function.

In MMD and LMMD, the feature mapping function uses multi-kernel Gaussian function, and the number of the Gaussian kernel is fixed at 5.

In multi-loss optimization training, the fixed hyperparameter *α*_*i*∈{*S*, *T*, *y*, *MMD*, *LMMD*}_ is {1, 1, 3, 1, 1}, {0.1, 0.1, 2, 1, 0.1}, {1, 1, 5, 2, 1}, {1, 1, 2, 1, 0.1}, {0.1, 0.1, 5, 1, 0.1}, and {0.1,0.1,5,2,0.1}, respectively. Under the six experimental schemes, the learning rate and batch size of TLMLDA model were 0.00001 and 100, respectively. TLMLDA uses an Adam optimizer and a SoftMax classifier, and the training epoch is set to 500.

We set up four ablation models: (a) TLMLDA_w is obtained by TLMLDA only deleting the fixed hyperparameter *α* in the dynamic weighting factor *w*_*i*_. (b) TLMLDA_*α* is obtained by TLMLDA only using the fixed hyperparameter *α* in the dynamic weighting factor *w*_*i*_. (c) TLMLDA_L and TLMLDA_M are obtained by TLMLDA only using LMMD and MMD, respectively.

In addition, some state-of-the-art cross-corpus SER methods are used as comparison methods, domain adaptive subspace learning (DoSL) [14], transfer sparse discriminant subspace learning (TSDSL) [17], joint distribution adaptive regression (JDAR) [18], and deep belief network and back propagation (DBN + BP) [28]. Meanwhile, PCA + SVM was selected as the benchmark method for comparison experiments, and the support vector machine (SVM) classifier uses linear kernel function and penalty coefficients searching in {0.001,0.01,0.1,1,10}.

Finally, we report the accuracy of emotion recognition by using the weighted average recall (WAR). WAR is defined as the average of the test accuracy of all samples.

### 3.3. Ablation Experiments

The recognition results of the TLMLDA and four ablation experiments are illustrated in Table 3.

The results of TLMLDA are significantly better than those of the ablation experimental methods. Compared with TLMLDA_w and TLMLDA_*α*, the average WAR of TLMLDA is improved by 6.21% and 6.54% in six cross-corpus SER experimental schemes. This is because TLMLDA training uses both fixed hyperparameter and dynamic weights, which prevent model training offset and disordered feature transfer. As a result, TLMLDA has better performance in cross-corpus SER.

Compared with TLMLDA_L and TLMLDA_M, TLMLDA still has made significant improvements. This is because TLMLDA_L and TLMLDA_M only perform global domain feature alignment or subdomain feature alignment, and some information is missing. Therefore, the recognition results of these two algorithms are inferior to those of TLMLDA.

**Figure 2:**
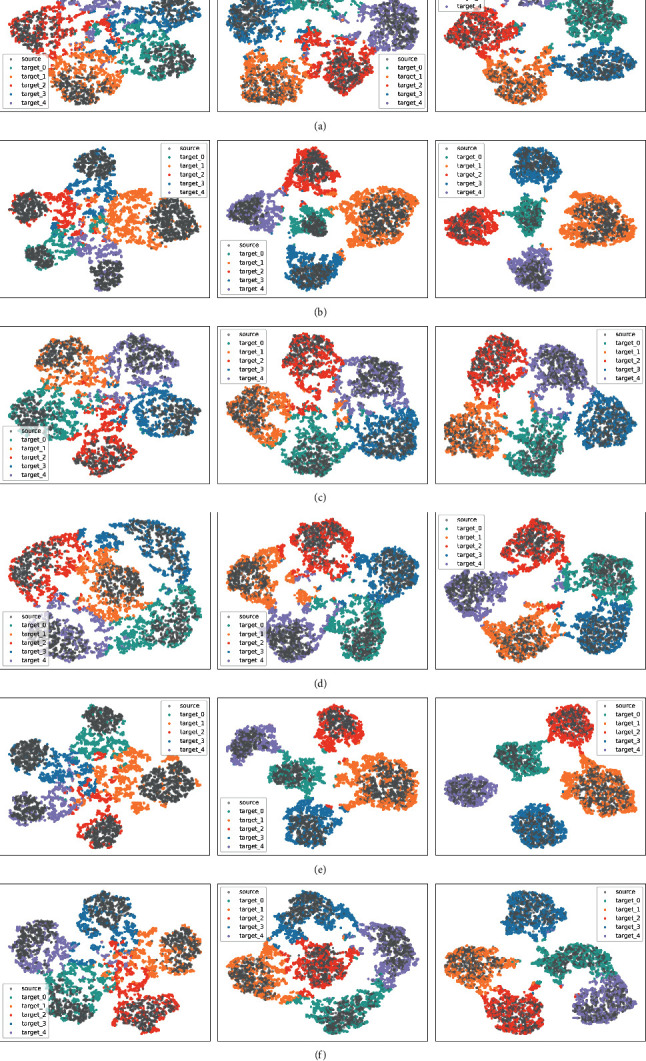
The t-SNE visualization of feature distributions (left: Only_cls, mid: TLMLDA_M, and right: TLMLDA). (a) E⟶B, (b) B⟶E, (c) E⟶C, (d) C⟶E, (e) B⟶C, and (f) C⟶B.

To further evaluate the performance of TLMLDA, we use the t-SNE method [40] to visualize the feature distributions of six cross-corpus tasks after using TLMLDA and TLMLDA_M. To better observe the effect of the domain adaptation process, we also compare the feature distribution maps obtained without using domain adaptation method, that is, using only the source domain classification loss (Only_cls), on the left side, as shown in Figure 2 and Figures 2(a)–2(f), the feature distribution maps from left to right are obtained by Only_cls, TLMLDA_M, and TLMLDA, respectively, and the source domain samples are marked in gray. It can be noted that, in the leftmost image, although the source domain can be well classified, the model obtained by training only with the source domain classification loss cannot discriminate the target domain sample features well, and after performing feature distribution alignment using MMD, it can improve the discrimination of the target domain features, but there are still some cases of class mismatching, and in order to observe the effect of subdomain adaptation, the target domain samples are performed based on the predicted class coloring, and the performance of the algorithm is evaluated by observing the proximity of the target domain samples to the source domain samples. The visualization results reveal some important observations; as analyzed above, in the feature distribution map obtained by executing TLMLDA_M, there is a serious subdomain mismatch problem, several target subdomains cannot be well aligned with the source domain, and a large number of target domain samples are confused in several category centers, leading to poor classification results. In TLMLDA, the problem of subdomain sample mismatch is greatly alleviated, and the low-resolution samples in the center of the feature distribution map are further migrated to the source domain. The visualization results show that the TLMLDA algorithm can better complete the feature distribution alignment process and learn better feature representations, which proves the effectiveness of the algorithm.

## 4. Comparison with Other Algorithms

The recognition results of the TLMLDA, benchmark method, and some state-of-the-art methods are illustrated in Table 4.

First, it is clear from the results that TLMLDA obtains the best overall performance among all methods in most scenarios. Compared with several other methods, it can be demonstrated that TLMLDA achieves significant improvements in most tasks. In particular, the WAR in E⟶B is 8.08%∼29.07% ahead of other algorithms. Compared with the best baseline method DoSL, the average WAR improves by 3.88%, and the WAR improves by 0.2%∼29.07% compared with other algorithms in the five settings of E⟶B, B⟶E, E⟶C, B⟶C, and C⟶B. These results indicate that TLMLDA improves the domain invariant representation of features with more robust generalization during cross-corpus speech emotion recognition.

Second, it can be observed that those algorithms (DoSL, TSDSL, and JDAR) that used transfer learning method all outperformed the no-transfer algorithms (PCA + SVM, DBN + BP), which validates the challenge of cross-corpus speech recognition introduced in the previous section; that is, if the source and target domains are from different corpora, a model that is trained on the source domain and performs well will have a dramatic degradation in performance on the target domain, which proves that the different distribution of data features in different corpora impairs the generalizability of the model, and adding transfer learning methods to the traditional speech emotion recognition framework is an effective solution.

Finally, excluding baseline methods that do not use transfer learning, it can be observed in Table 4 that TLMLDA also achieves significant performance improvements compared to transfer learning-based algorithms DoSL, TSDSL, and JDAR. Compared with DoSL and TSDSL, which are algorithms that consider only global transfer, TLMLDA jointly considers the alignment of global and local feature distributions, which can well maximize the interclass distance and minimize the intraclass distance to improve the model generalization ability. Compared with JDAR, although JDAR jointly considers the edge probability distribution and conditional probability distribution between the source and target domain corpora, both of which contribute to model learning domain invariant representation, JDAR does not dynamically adjust the weights between multiple losses; when applied in practice and at different training stages, marginal probability distributions and conditional probability distributions may contribute differently to the discrepancy in feature distributions; therefore, TLMLDA with the addition of dynamically adjusted loss weights obtains a superior performance.

### 4.1. Time and Space Complexity of TLMLDA

In TLMLDA, the features of an input batch first pass through the three hidden layers of the encoder and then enter the three hidden layers of the decoder to recover the original dimensions in order to calculate the reconstruction loss; at the same time, the features learned by the encoder are classified after the fully connected layers, and the features in the source and target domains enter both MMD and LMMD for alignment. Since the reconstruction process and the computation of reconstruction loss are executed in parallel with the fully connected layers, the computation of MMD, LMMD, and the time complexity should be taken as the larger computation between the two process. In auto-encoder and DNN, the computation can be expressed as the input dimension of the features multiplied by the output dimension, the dimension of the matrix computation performed in MMD and LMMD is the batch size, and the computation of the reconstruction loss is the batch size multiplied by the original feature dimension. In addition, the bias in the neural network also needs to be considered when calculating the space complexity, so the time and space complexity of TLMLDA can be expressed as follows:

(7) $$\begin{array}{c} TIME∼O\left[B\left(\overset{3}{\underset{i=1}{\sum }}H_{i_{in}}H_{i_{out}}+\max\;\left(\overset{2}{\underset{i=1}{\sum }}F_{i_{in}}F_{i_{out}}+B^{2},\overset{3}{\underset{i=1}{\sum }}H_{i_{in}}H_{i_{out}}+F\right)\right)\right]\text{,}\\ SPACE∼O\left(\overset{3}{\underset{i=1}{\sum }}\left(H_{i_{in}}+1\right)H_{i_{out}}+\overset{2}{\underset{i=1}{\sum }}\left(F_{i_{in}}+1\right)F_{i_{out}}\right)\text{.} \end{array}$$

In formulas (7), *B* is the batch size and also the matrix dimension for MMD and LMMD calculations, *H*_*i*_*in*_, *H*_*i*_*out*_, *F*_*i*_*in*_, *F*_*i*_*out*_ represent the input dimension and output dimension of hidden layer *i* and fully connected layer *i*, respectively, and F denotes the original feature dimension.

## 5. Conclusions

In this paper, we have proposed a TLMLDA model to deal with the cross-corpus SER problem. Firstly, TLMLDA uses the DAE network to compress redundant information, and then the powerful nonlinear fitting ability of DNN is used to further learn low-dimensional emotional features. Secondly, TLMLDA measures the feature distribution distances of the source and target domains from the global and subdomain perspectives simultaneously. Lastly, TLMLDA constructed a multi-loss dynamic adjustment algorithm to train the model, which helps to improve the model recognition ability. Based on experimental results, it is clear that our proposed TLMLDA can effectively improve the cross-corpus SER performance. Furthermore, the proposed method has some limitations, such as the computational power consumed in considering the global and local alignment process. Despite the excess performance of the devices now, developing a metric that can accomplish both global and local alignment will be a great boost to the practical application of SER. In addition, the lack of a well-performing end-to-end speech emotion recognition system is one of the current obstacles to the implementation of SER applications. Therefore, our subsequent work will focus on the optimization of transfer learning algorithms and the development of end-to-end speech emotion recognition, which will be valuable for SER applications to achieve excellent performance in various fields.

## Figures and Tables

**Table 1:** A brief summary of related work.

References	Year	Methods	Features	Corpus
Zong et al. [13]	2016	Least squares regression	INTERSPEECH 2009	Berlin, AFEW 4.0, eNTERFACE
Liu et al. [14]	2018	Feature selection + SVM	INTERSPEECH 2009	Berlin, AFEW 4.0, eNTERFACE
Luo et al. [15]	2019	NMF + MMD	Segmental features	Berlin, CASIA, eNTERFACE, Estonian
Song [16]	2019	TLSL	INTERSPEECH 2010	Berlin, FAU-AIBO, eNTERFACE
Zhang et al. [17]	2020	TSDSL	INTERSPEECH 2010	Berlin, BAUM-1a, eNTERFACE
Zhang et al. [18]	2021	JDAR	INTERSPEECH 2010	Berlin, CASIA, eNTERFACE
Zehra et al. [19]	2021	Ensemble learning	Spectral and prosodic	SAVEE, UrduRDU, EMO-DB, EMOVO
Latif et al. [28]	2018	DBNs	eGeMAPS feature set	FAU-AIBO, SAVEE IEMOCAP, EMO-DB, EMOVO
Zhang et al. [29]	2019	Deep metric learning	Log Mel-frequencyfilter-bank energy	IEMOCAP, MSP-improv
Ahn et al. [30]	2021	Few-shot learning	INTERSPEECH 2010	IEMOCAP, CREMA-D, MSP-IMPROV,Berlin, Korean multimodal emotion dataset
Chang et al. [31]	2021	Adversarial learning	INTERSPEECH 2010	IEMOCAP, MSP-improv, MSP-PODCAST
Sneha et al. [32]	2022	VAE with KL annealing	eGeMAPS feature set	IEMOCAP, SAVEE, Berlin, CaFE, URDU, AESD

**Table 2:** Emotional labels and samples sizes selected for six cross-corpus SER schemes.

Schemes	Corpus	Emotional labels	Size
E⟶B	eNTERFACE Berlin	Anger, sad, fear, happy, disgust	1072
B⟶E			375
E⟶C	eNTERFACE CASIA	Anger, sad, fear, happy, surprise	1072
C⟶E			1000
B⟶C	Berlin CASIA	Anger, sad, fear, happy, neutral	408
C⟶B			1000

**Table 3:** Experimental results of the use of ablation experiments.

Algorithm	E⟶B	B⟶E	E⟶C	C⟶E	B⟶C	C⟶B	Average
TLMLDA_w	51.95	31.15	31.10	30.40	32.70	53.53	38.51
TLMLDA_*α*	46.62	34.33	31.60	30.67	32.70	53.13	38.18
TLMLDA_L	36.76	21.12	28.70	28.01	20.05	42.71	29.56
TLMLDA_M	54.08	38.28	34.90	29.23	32.70	54.33	40.58
**TLMLDA**	**58.93**	**43.16**	**35.40**	**32.74**	**41.00**	**57.11**	**44.72**

The bold values are the highest recognition rate in each task to reflect the rationality of the TLMLDA model, because TLMLDA has obtained the best performance compared with other ablation experimental models.

**Table 4:** Experimental results of the use of other algorithms.

Algorithm	E⟶B	B⟶E	E⟶C	C⟶E	B⟶C	C⟶B	Average
PCA + SVM	50.85	33.48	28.40	27.61	33.13	43.38	36.14
DoSL [14]	50.55	33.03	35.20	**33.81**	39.23	53.20	40.84
TSDSL [17]	47.41	35.44	32.50	33.25	37.40	56.74	40.46
JDAR [18]	48.74	38.14	30.30	28.43	38.60	49.58	38.97
DBN + BP [28]	29.86	32.21	24.20	31.02	35.80	49.59	33.78
**TLMLDA**	**58.93**	**43.16**	**35.40**	32.74	**41.00**	**57.11**	**44.72**

The bold values are the highest recognition rate in each task to reflect the rationality of the TLMLDA model, because TLMLDA has obtained the best performance compared with other ablation experimental models.

## Data Availability

The data used to support the findings of this study are available from the corresponding author upon request.
